# Intravesical administration of exogenous microRNA-145 as a therapy for mouse orthotopic human bladder cancer xenograft

**DOI:** 10.18632/oncotarget.4129

**Published:** 2015-05-12

**Authors:** Teruo Inamoto, Kohei Taniguchi, Kiyoshi Takahara, Ayako Iwatsuki, Tomoaki Takai, Kazumasa Komura, Yuki Yoshikawa, Taizo Uchimoto, Kenkichi Saito, Naoki Tanda, Junko Kouno, Koichiro Minami, Hirofumi Uehara, Hajime Hirano, Hayahito Nomi, Satoshi Kiyama, Yukihiro Akao, Haruhito Azuma

**Affiliations:** ^1^ Department of Urology, Osaka Medical College, Osaka, Japan; ^2^ United Graduate School of Drug Discovery and Medical Information Sciences, Gifu University, Gifu, Japan

**Keywords:** microRNA-145, intravesical instillation, bladder cancer

## Abstract

We previously reported that the level of microRNA (miR)-145 is attenuated in human bladder cancer cells. In this current study, we investigated whether intravesical administration of miR-145 could be a potential therapeutic strategy for controlling bladder cancer by using an orthotopic human bladder cancer xenograft model. Following transfection of 253J B-V cells with miR-145, the effects of the ectopic expression of miR-145 were examined by performing MTT, Western blotting analysis, Hoechst33342 staining, and wound healing assay *in vitro*. Also, a mouse orthotopic human bladder cancer model was established by inoculating 253J B-V cells into the bladder wall of mice. The anti-cancer effects of intravesical injections of miR-145 into these mice were then assessed. Transfection of 253J B-V cells with miR-145 induced apoptosis and suppression of cell migration *in vitro*. Western blotting showed that the levels of c-Myc, socs7, FSCN1, E-cadherin, β-catenin, and catenin δ-1 were decreased and that the PI3K/Akt and Erk1/2 signaling pathways were increased in compensatory fashion. *In vivo*, mice treated with miR-145 showed 76% inhibition of tumor growth, with a significant prolongation of animal survival (*p* = 0.0183 *vs.* control). Western blotting showed that both apoptosis and cell motility-related genes were significantly decreased as seen *in vitro*. Furthermore, PI3k/Akt and Erk1/2 signaling pathways, which were activated in a compensatory manner *in vitro*, were decreased *in vivo.* Intravesical administration of exogenous miR-145 was thus concluded to be a valid therapy for bladder cancer in this human bladder cancer xenograft model.

## INTRODUCTION

In 2012 more than 73000 new cases of bladder cancer were reported in the United States, along with 14,880 deaths due to this disease. Without treatment, the prognosis of locally advanced or metastatic bladder cancers is dismal. Increasing efforts should thus be made to offer patients with localized bladder cancer a rigid therapy with the aim of minimizing recurrence and progression. At present, the standard therapy for non-muscle invasive bladder cancer (NMIBC) is an intra-vesical BCG therapy to prolong the time of recurrence and progression. Even though most NMIBC patients do not die of bladder cancer, the majority of them must endure recurrence and high-risk advancement of their cancer. Chamie *et al*. investigated Surveillance, Epidemiology, and End Results (SEER)-Medicare data and found that nearly 75% of patients with high-risk NMIBC suffer recurrence and progression of their cancer or die within 10 years of their diagnosis [1]. Hence, a significant proportion of high-risk NMIBC patients is not suited for transurethral resection of the bladder tumor (TUR-Bt) plus BCG therapy.

MiR-145 is one of the representative anti-oncomiRs in various cancers. Previously, we reported that miR-145 is down-regulated and acts as a tumor suppressor in colon adenomas [2, 3], colon cancers [4], gastric cancers [5], chronic lymphocytic leukemias and B-cell lymphomas [6], several cancer cell lines [7-9], and of course in bladder cancer cells [10, 11]. Furthermore, we found that miRs-143 and -145, which are transcribed as the same primary miRNA at chromosome 5q33, are down-regulated in most of cancer cell lines and tissues [12]. Moreover, we found that down-regulation of miR-145, which is caused by extracellular disposal via microvesicles, is related to 5-FU resistance in colon cancer cells [13]. These findings suggested that miR-145 has the potential to be an anti-cancer drug against various cancers including bladder cancer.

However, to utilize microRNA in the clinical setting, the route of its administration is a very important consideration. Because microRNAs can be easily and rapidly degraded in the circulating blood flow, administration of microRNAs by oral or intravenous administration is not the ideal way for drug delivery. For realization of an RNA medicine, various kinds of Drug Delivery System (DDS), such as liposomes and against nuclease and side effects have been applied. However, no proper DDS has been established yet. To avoid serious general side effects, bladder instillation is an apparently better way for microRNA to reach diseased tissue, where bladder cancer arises from the epithelial lining even in liposome-encapsulated miR-145. In the present study, we evaluated the potency of miR-145 as a therapeutic agent for intravesical therapy, using our established mouse orthotopic bladder cancer xenograft model [14, 15].

## RESULTS

### Exogenous miR-145 reduces cellular viability of human bladder 253J B-V cells

**Figure 1:**
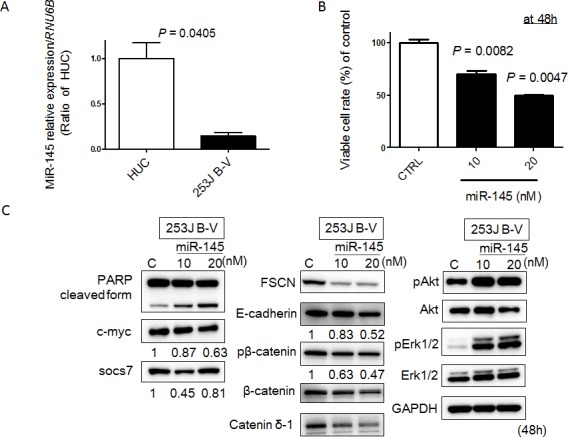
MiR-145 acts as a tumor suppressor in 253 J B-V cells **A.** Relative expression levels of miR-145 in HUC and 253 J B-V cells. **B.** Effects of ectopic expression of miR-145 on cell viability. The growth was monitored by performing the MTT assay and shown as a percentage of the control. **C.** Expression of various proteins estimated by Western blot analysis at 48 h after transfection of 253 J B-V cells with miR-145. The concentration of miR-145 was 10 or 20 nM in each experiment.

Previously, we established a mouse orthotopic bladder cancer model using 253J B-V cells [14, 15]. In this current study, we validated whether miR-145 would have anti-tumor effects in a xenografted mouse. Firstly, however, we examined the *in vitro* anti-tumor effects of miR-145 on 253J B-V cells, as previously observed in various cancer cells including bladder cancer ones [8-11]. As a result, the expression level of miR-145 in 253J B-V cells was markedly down-regulated compared with that in normal human urothelial cells (HUC; Figure 1A). Transfection of human 253J B-V cells with miR-145 resulted in a pronounced growth suppression that occurred in a dose-dependent fashion (Figure 1B). These analyses demonstrated that transfection with miR-145 was an effective way to accomplish growth suppression in 253J B-V cells.

### Exogenous miR-145 induces apoptosis in and inhibits cell migration of 253J B-V cells

**Figure 2:**
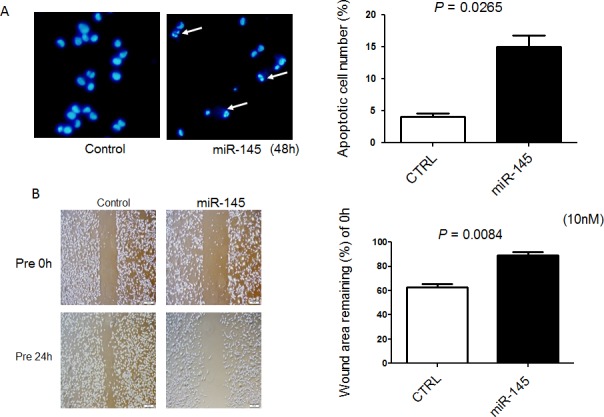
Exogenous miR-145 induces apoptosis in and inhibits cell migration of 253J B-V cells **A.** Hoechst 33342 staining at 48 h after transfection of 253 J B-V cells with miR-145 at a concentration of 20 nM. Apoptotic cells are indicated by the white arrows. **B.** Cells were also analyzed for motility by using wound healing assay after transfection of 253 J B-V cells with miR-145 at a concentration of 10 nM. Cells were photographed and counted by use of an imaging system. The initial (0 h) and the residual gap widths, assessed 24 h after wounding, were determined from photomicrographs.

Recently, we found that ectopic expression of miR-145 induces apoptosis through the down-regulation of c-Myc and socs7 in bladder cancer cells [10, 11]. Therefore, we examined 253J B-V cells by Western blotting analysis and Hoechst33342 staining after transfection of them with miR-145. As a result, Western blotting analysis clearly showed the cleaved form of PARP and down-regulation of c-Myc and socs7 (Figure 1C). Hoechst33342 nuclear staining indicated a typical apoptotic appearance such as condensed chromatin and nuclear fragmentation in the miR-145-treated 253J B-V cells (Figure 2A). On the other hand, Western blotting showed that PI3K/Akt and Erk1/2 signaling pathways had been activated as compensatory signaling pathways (Figure 1C). Also, recently, we found that miR-145 inhibits cell migration through the down-regulation of Fascin-1 (FSCN1), E-cadherin, β-catenin, and catenin δ-1 in various cancer cells [8, 9]. Therefore, we performed a wound healing assay and assessed the protein expression levels of these cell migration related-genes by Western blot analysis. As shown in Figure 2B, the ectopic expression of miR-145 significantly decreased cell migration of 253J B-V cells approximately 30%. Western blotting analysis showed that the expression levels of all FSCN1, E-cadherin, β-catenin, and catenin δ-1 were also significantly down-regulated in the miR-145-treated 253J B-V cells (Figure 1C). These findings taken together suggested that miR-145 had the same anti-tumor effects in 253J B-V cells as previously found in other cancer cells [8, 9, 11]. We concluded that 253J B-V cells would be suitable for validating the anti-tumor effects of miR-145 in our mouse orthotopic human bladder cancer xenograft model.

### Inhibition of tumor growth with prolonged animal survival in an orthotopic human bladder cancer xenograft model in nude mice

**Figure 3:**
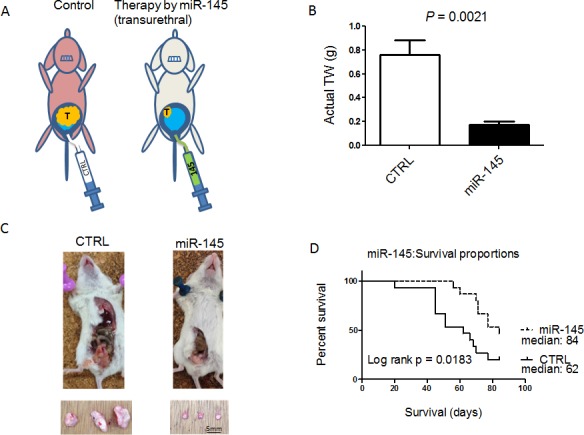
Intravesically administered miR-145 inhibits orthotopic bladder tumor growth *in vivo* **A.** Schema of our mouse orthotopic bladder cancer model. Intravesical growth of bladder cancer in this orthotopic mouse model was established. Transurethral administration of miR-145 prevented the intravesical growth of bladder cancer. **B.** The anti-tumor effect of miR-145 was analyzed after intravesical administration of miR-145 to established 253 J B-V tumors. Average tumor weights were determined. **C.** Representative photographs of xenografted tumors *in situ* ( upper photos) and excised (lower photos) from mice. **D.** Survival of 8W old sex-matched mice that had been inoculated with bladder cancer 253J B-V cells (2 × 10^5^) implanted into the bladder wall of nude mice was estimated by the Kaplan-Meier method, and statistical significance was calculated by using the log-rank test. *n* = 15 mice for each group.

To examine the growth inhibitory effect of miR-145 on *in vivo* growth of bladder tumors, we used our mouse model bearing 253J B-V bladder xenografted tumors that had been established by transplanting the tumor cells orthotopically in the bladder wall of nude mice [14, 15] (Figure 3A). After the treatment with miR-145, the mice were sacrificed; and the tumor volumes were then measured. As a result, miR-145 significantly inhibited the tumor growth of orthotopic 253J B-V xenografts when used at a dose of 100 nM (Figure 3B and 3C). When compared with the untreated group, the mice treated with miR-145 showed 76% inhibition of tumor growth (Figure 3B and 3C, *P* = 0.0021). The effects of intravesical injection of miR-145 on animal survival were evaluated by preparing Kaplan-Meier plots, with the survival duration analyzed for significance by performing log-rank survival analysis. The treatment with miR-145 significantly prolonged animal survival in mice bearing orthotopic 253J B-V xenografts when compared with that for the controls (Figure 3D, *p* = 0.0183).

### MiR-145 can reach xenografted tumors by intravesical injection

**Figure 4:**
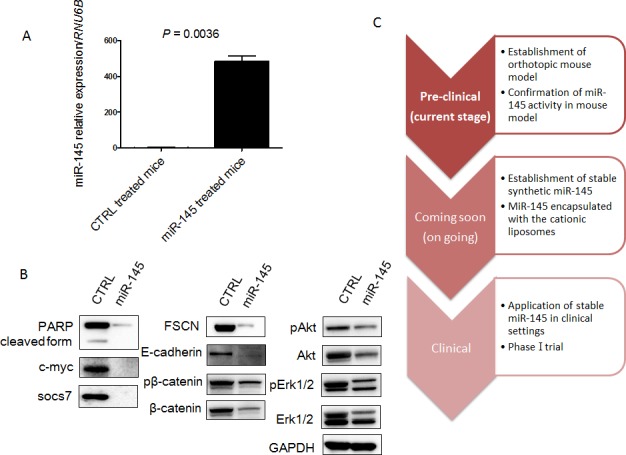
MiR-145 can reach xenografted tumors by intravesical injection and assessment of protein expression of miR-145-targeted and related genes in the xenografted tumor samples **A.** Relative expression levels of miR-145 in xenografted tumors treated with miR-145 or control miR. **B.** Expression of various proteins in the xenografted tumors as estimated by Western blot analysis. **C.** Schematic diagram of current and future studies. In this study, we showed that miR-145 could reach xenografted tumors by intravesical injection and have anti-tumor effects in our mouse model. We are comparing the anti-tumor effects between BCG and miR-145 in our mouse model now and plan to begin a clinical phase-one trial by using this system in the near future.

Next, to validate the growth suppression of xenografted tumor by intravesical injection of miR-145, we examined the expression level of miR-145 in xenografted tumors by qRT-PCR and the expression levels of possible miR-145-targeted genes by Western blotting analysis. Expectedly, the expression level of miR-145 was significantly up-regulated in the xenografted tissues compared with that in the control group (Figure 4A). Western blotting analysis showed that the protein levels of miR-145-targeted genes such as c-Myc, socs7, FSCN1, E-cadherin, and β-catenin were significantly down-regulated (Figure 4B). Interestingly, c-Myc and socs7 were down-regulated to a greater extent compared with the results of the *in vitro* experiments. The cleaved form of PARP was not up-regulated because the proform of PARP had almost disappeared (Figure 4B). Furthermore, the compensatory activation of PI3K/Akt and Erk1/2 signaling pathways, which were observed *in vitro*, were also suppressed (Figure 4B). These findings taken together suggested that the intravesical administration of miR-145 effectively exhibited its anti-tumor effect in the xenografted tumor through overcoming the serious immunological side effects caused by liposome-encapsulated miRNA.

## DISCUSSION

Especially with regards to the treatment of bladder cancer, an intensive focus has been to identify therapeutic approaches that can fine tune the suppression of growth of host bladder cancer [11, 17]. The intravesical approach for the delivery of therapeutic agents has been used in clinical settings as a promising and attractive therapeutic method for non-muscle invasive bladder cancer [18, 19]. In the current study, we focused on investigating the practical aspects of miR-145 therapy of human bladder cancer. Because miRNA is unstable and rapidly degraded by ribonuclease (RNase) in the body, miRNA therapy would not seem to be practical for any neoplasm. In this context, the bladder is an organ that therapeutic miRNA can be imagined to reach easily by the trans-urethal approach. Koga *et al.* investigated the stability of miRNA in feces [20]. They added RNase to culture media of HT-29 cells and fecal homogenates and then analyzed the relative amount of miRNA by real-time RT-PCR. They found that cellular miRNA or exosomal miRNAs were protected from RNase by the cellular membrane or the exosome; meanwhile, free miRNA was degraded immediately and completely by the RNase. Therefore, miRNAs are usually delivered in encapsulated forms such as liposomes. Even in liposome-encapsulated forms, however, they are degraded in part by RNase or immune surveillance by cells such as macrophages.

We conducted intravesical instillation of liposome-encapsulated miR-145 after emptying the residual urine from the bladder. By doing so, we avoided the effects of RNase and immunological surveillance systems induced by liposomes as a type of DDS (Figures 3 and 4). Furthermore, we showed an additional anti-tumor mechanism, i.e., suppression of compensatory molecular pathways, by continuous intravesical instillation therapy with miR-145 achieved with 8 periods of administration (Figure 4B) as opposed to the increased activity of these compensatory pathways found *in vitro* (Figure 1C). In our experiment, the activation of PI3K/Akt and Erk1/2 signaling pathways were observed *in vitro.* Although, these pathways were suppressed *in vivo*. Mitogen-activated protein kinase (MAPK) cascades are key signaling pathways involved in the regulation of normal cell proliferation, survival and differentiation [21]. Aberrant regulation of MAPK cascades contribute to cancer and other human diseases [21]. Also, chemical mediators, including MAPK, act dynamically *in vivo*, showing the different activation observed *in vitro*. To date, the actions of each chemical mediator have been demonstrated *in vitro*, how such chemical mediators act cooperatively or counteractively *in vivo* remains largely unknown [22]. Examination of the present study revealed PI3K/Akt and Erk1/2 activation *in vitro* and an opposite profile *in vivo*. These results underscore complementary roles of the PI3K/Akt and Erk1/2 pathways for bladder cancer proliferation and motility *in vitro* and raise implications for a solid inhibitory effect of these signaling pathways induced by intra-vesical miR-145 *in vivo*.

With practical BCG therapy, liquid BCG is introduced through a catheter directly into the bladder rather than giving it orally or otherwise intravenously. Likewise, miR-145 given this way will mainly affect the cells lining the inside of the bladder, with little to no effect on cells elsewhere. This means that any other organs outside of the bladder lining are not affected by the drug, thus minimizing potential adverse events triggered by the therapeutic miRNA. It is known that intravesical BCG therapy has major adverse side effects such as dysuria and frequency [18]. Therefore, we regard that miR-145 therapy would be more suitable for patients when we consider the side effects. Now, we are comparing the anti-tumor effects between BCG and miR-145 therapy in our mouse model (Figure 4C). Also, to further enhance the anti-tumor effects, we are making chemically-modified miR-145 that can possibly be given in its naked form. We consider that clinical application of miR-145 therapy may be realized in the near future by combining present knowledge with new techniques.

In conclusion, the present findings for the first time showed that miR-145 was an effective tumor suppressor miRNA against bladder cancer and that its intravesical delivery effectively inhibited the tumorigenic phenotypes without any adverse effects. Thus we propose that the use of miR-145 by the intravesical approach can become a successful therapeutic strategy for bladder cancer.

## MATERIALS AND METHODS

### Cell culture and cell viability

Cells of the human bladder cancer cell line 253J B-V were cultured in RPMI-1640 medium supplemented with 10% (v/v) heat-inactivated FBS (Sigma-Aldrich Co, St. Louis, MO, USA) and 2 mM L-glutamine under an atmosphere of 95% air and 5% CO_2_ at 37°C. Cells (5 × 10^3^) were plated in 96-well plates for 24 hours and then incubated for 48 hours with or without miR-145 at various concentrations for a total volume of 100 μL. We measured the effect of miR-145 on cell growth by use of an MTT Cell Growth Assay Kit (MERCK MILLIPORE). In brief, tetrazolium salt was added to each well; and after 2 hours of incubation, the amount of water-soluble formazan dye formed by bioreduction in the presence of an electron carrier, 1-methoxy-5-methylphenazinium, was measured at 450 nm by using a microplate reader (Bio-Rad, Hercules, CA). All samples were tested in triplicate. Values reported were presented as the mean of triplicate wells, and the SE was within 15%.

### Transfection experiments

253J B-V cells were seeded in 6-well plates at a concentration of 0.5 × 10^5^ per well (10-30% confluence) on the day before the transfection. The mature type of miR-145 (mirVana™ miRNA mimic; Ambion, Foster City, CA, USA) was used for the transfection of the cells, which was achieved by using cationic liposomes, Lipofectamine™ RNAiMAX (Invitrogen), according to the manufacturer's Lipofection protocol. The nonspecific control miRNA (HSS, Hokkaido, Japan) sequence was 5′-GUAGGAGUAGUGAAAGGCC-3′, which was used as a control for nonspecific effects [3] [9]. The sequence of the mature type of miR-145 used in this study was 5′-GUCCAGUUUUCCCAGGAAUCCCUU-3′. The effects manifested by the introduction of miR-145 into the cells were assessed at 48 h after the transfection.

### Western blotting

Protein extraction and Western blotting experiments were performed as described in our previous reports [9] [10]. Primary antibodies used were as follow: anti-socs7 (Santa Cruz Biotechnology, Santa Cruz, CA, USA); antibodies against PARP, pAkt, Akt, pErk1/2, Erk1/2, c-Myc, E-cadherin, pβ-catenin, β-catenin, cateninδ-1, and GAPDH (Cell Signaling Technology, Inc., Danvers, MA, USA); and anti-FSCN1 (Abcam, Cambridge, UK). HRP-conjugated goat anti-rabbit and horse anti-mouse IgG (Cell Signaling Technology) were used as secondary antibodies. GAPDH served as an internal control.

### Real-time reverse transcription-PCR

Total RNA was isolated from cultured cells or tumor tissues by using a NucleoSpin miRNA isolation kit (TaKaRa, Otsu, Japan). RNA concentrations and purity were assessed by UV spectrophotometry. RNA integrity was checked by formaldehyde gel electrophoresis. To determine the expression levels of miR-145, we conducted quantitative RT-PCR (qRT-PCR) by using TaqMan MicroRNA Assays (Applied Biosystems) and THUNDERBIRD Probe qPCR Mix (TOYOBO Co., LTD., Osaka, Japan) according to the manufacturer's protocol. *RNU6B* was used as an internal control. The relative expression levels were calculated by the ΔΔCt method.

### Hoechst33342 staining

253J B-V cells were collected at 48 h after transfection with miR-145 (20 nM). The details of the experimental protocol were given in a previous report [11, 16]. The number of apoptotic cells among 200 cells was counted.

### Wound healing assay

253J B-V cells were seeded into six-well plates at a concentration of 1.0 × 10^5^cells/well and transfected with miR-145 (10 nM). Details of the experimental protocol may be seen in our previous reports [8, 9]. The initial gap width (0 hour) and the residual gap width (24 hours) after the scratches had been made were calculated from photomicrographs.

### *In vivo* xenograft model

253J B-V cells (2 × 10^5^) were implanted into the bladder wall of female nude mice. Once tumors had developed (average volume 70 mm^3^), they were treated with 8 intravesical injections of miR-145. Synthetic miR-145 (100 nM) complexed with 100 μl of Lipofectamine^TM^ RNAiMAX reagent (Invitrogen) was added to 50 μl of PBS and delivered 8 times intravesically every other day. Actual tumor sizes were measured 4 weeks after implantation of the cancer cells. Effects of miR-145 on human bladder xenograft mice survival were evaluated by preparing Kaplan-Meier plots, and survival time of animals was analyzed for significance by log-rank survival analysis.

### Statistics

Each examination was performed in triplicate. Statistical significances of differences were evaluated by performing the two-sided Student's *t*-test. For *in vivo* experiments, we use the Kaplan-Meier method; and statistical significance was calculated by using a log-rank test. The values were presented as the mean ± standard deviation. A *P* value < 0.05 was considered to be statistically significant.

## SUPPLEMENTARY MATERIAL FIGURE



## References

[1] Chamie K, Litwin MS, Bassett JC, Daskivich TJ, Lai J, Hanley JM, Konety BR, Saigal CS, Urologic Diseases in America P (2013). Recurrence of high-risk bladder cancer: a population-based analysis. Cancer.

[2] Kamatani A, Nakagawa Y, Akao Y, Maruyama N, Nagasaka M, Shibata T, Tahara T, Hirata I (2013). Downregulation of anti-oncomirs miR-143/145 cluster occurs before APC gene aberration in the development of colorectal tumors. Medical molecular morphology.

[3] Akao Y, Nakagawa Y, Hirata I, Iio A, Itoh T, Kojima K, Nakashima R, Kitade Y, Naoe T (2010). Role of anti-oncomirs miR-143 and -145 in human colorectal tumors. Cancer gene therapy.

[4] Akao Y, Nakagawa Y, Naoe T (2007). MicroRNA-143 and -145 in colon cancer. DNA and cell biology.

[5] Takagi T, Iio A, Nakagawa Y, Naoe T, Tanigawa N, Akao Y (2009). Decreased expression of microRNA-143 and -145 in human gastric cancers. Oncology.

[6] Akao Y, Nakagawa Y, Kitade Y, Kinoshita T, Naoe T (2007). Downregulation of microRNAs-143 and -145 in B-cell malignancies. Cancer science.

[7] Akao Y, Nakagawa Y, Naoe T (2006). MicroRNAs 143 and 145 are possible common onco-microRNAs in human cancers. Oncology reports.

[8] Noguchi S, Mori T, Hoshino Y, Yamada N, Nakagawa T, Sasaki N, Akao Y, Maruo K (2012). Comparative study of anti-oncogenic microRNA-145 in canine and human malignant melanoma. The Journal of veterinary medical science / the Japanese Society of Veterinary Science.

[9] Yamada N, Noguchi S, Mori T, Naoe T, Maruo K, Akao Y (2013). Tumor-suppressive microRNA-145 targets catenin delta-1 to regulate Wnt/beta-catenin signaling in human colon cancer cells. Cancer letters.

[10] Noguchi S, Yasui Y, Iwasaki J, Kumazaki M, Yamada N, Naito S (2013). Replacement treatment with microRNA-143 and -145 induces synergistic inhibition of the growth of human bladder cancer cells by regulating PI3K/Akt and MAPK signaling pathways. Cancer letters.

[11] Noguchi S, Yamada N, Kumazaki M, Yasui Y, Iwasaki J, Naito S, Akao Y (2013). socs7, a target gene of microRNA-145, regulates interferon-beta induction through STAT3 nuclear translocation in bladder cancer cells. Cell death & disease.

[12] Iio A, Nakagawa Y, Hirata I, Naoe T, Akao Y (2010). Identification of non-coding RNAs embracing microRNA-143/145 cluster. Molecular cancer.

[13] Akao Y, Khoo F, Kumazaki M, Shinohara H, Miki K, Yamada N (2014). Extracellular disposal of tumor-suppressor miRs-145 and -34a via microvesicles and 5-FU resistance of human colon cancer cells. International journal of molecular sciences.

[14] Black PC, Brown GA, Dinney CP, Kassouf W, Inamoto T, Arora A, Gallagher D, Munsell MF, Bar-Eli M, McConkey DJ, Adam L (2011). Receptor heterodimerization: a new mechanism for platelet-derived growth factor induced resistance to anti-epidermal growth factor receptor therapy for bladder cancer. The Journal of urology.

[15] Inamoto T, Papineni S, Chintharlapalli S, Cho SD, Safe S, Kamat AM (2008). 1,1-Bis(3′-indolyl)-1-(p-chlorophenyl)methane activates the orphan nuclear receptor Nurr1 and inhibits bladder cancer growth. Molecular cancer therapeutics.

[16] Kumazaki M, Noguchi S, Yasui Y, Iwasaki J, Shinohara H, Yamada N, Akao Y (2013). Anti-cancer effects of naturally occurring compounds through modulation of signal transduction and miRNA expression in human colon cancer cells. The Journal of nutritional biochemistry.

[17] Fahmy M, Mansure JJ, Brimo F, Yafi FA, Segal R, Althunayan A, Hicks J, Meeker A, Netto G, Kassouf W (2013). Relevance of the mammalian target of rapamycin pathway in the prognosis of patients with high-risk non-muscle invasive bladder cancer. Human pathology.

[18] Witjes JA, Palou J, Soloway M, Lamm D, Kamat AM, Brausi M, Persad R, Buckley R, Colombel M, Bohle A (2013). Current clinical practice gaps in the treatment of intermediate- and high-risk non-muscle-invasive bladder cancer (NMIBC) with emphasis on the use of bacillus Calmette-Guerin (BCG): results of an international individual patient data survey (IPDS). BJU international.

[19] Sternberg IA, Dalbagni G, Chen LY, Donat SM, Bochner BH, Herr HW (2013). Intravesical gemcitabine for high risk, nonmuscle invasive bladder cancer after bacillus Calmette-Guerin treatment failure. The Journal of urology.

[20] Koga Y, Yasunaga M, Moriya Y, Akasu T, Fujita S, Yamamoto S, Matsumura Y (2011). Exosome can prevent RNase from degrading microRNA in feces. Journal of gastrointestinal oncology.

[21] Roberts PJ, Der CJ (2007). Targeting the Raf-MEK-ERK mitogen-activated protein kinase cascade for the treatment of cancer. Oncogene.

[22] Mizuno R, Kamioka Y, Kabashima K, Imajo M, Sumiyama K, Nakasho E, Ito T, Hamazaki Y, Okuchi Y, Sakai Y, Kiyokawa E, Matsuda M (2014). In vivo imaging reveals PKA regulation of ERK activity during neutrophil recruitment to inflamed intestines. The Journal of experimental medicine.

