# Supplementary material for: Intravesical administration of exogenous microRNA-145 as a therapy for mouse orthotopic human bladder cancer xenograft
Source: Oncotarget. 2015 May 12;6(25):21628–35. doi: 10.18632/oncotarget.4129 (PMC4673291; doi:10.18632/oncotarget.4129)
Supplement: Supplementary file 1 [file oncotarget-06-21628-s001.pdf]

Intravesical administration of exogenous microRNA-145 as a therapy for mouse orthotopic human bladder cancer xenograft

Supplementary Material

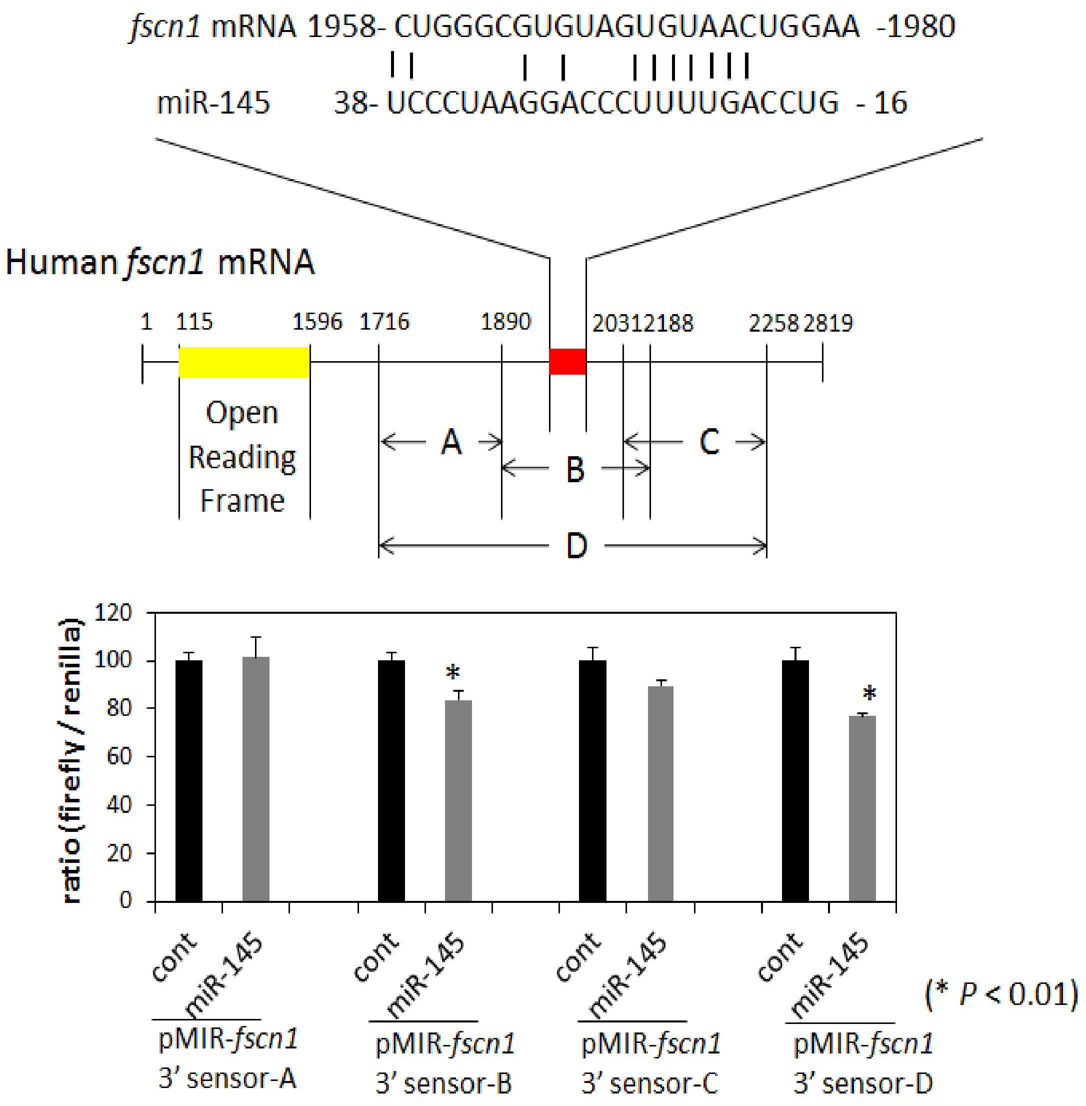

Supplemental Figure 1: MiR-145, which have conserved sequences in the 3'UTR of FSCN1 (actin-binding protein, Fascin homolog, inhibited FSCN1 expression. The signal from a luciferase reporter assay was significantly decreased at 2 miR-145 target sites, suggesting miR-145 directly regulates FSCN1.
